# Whole Transcriptome RNA-Seq Analysis of Breast Cancer Recurrence Risk Using Formalin-Fixed Paraffin-Embedded Tumor Tissue

**DOI:** 10.1371/journal.pone.0040092

**Published:** 2012-07-13

**Authors:** Dominick Sinicropi, Kunbin Qu, Francois Collin, Michael Crager, Mei-Lan Liu, Robert J. Pelham, Mylan Pho, Andrew Dei Rossi, Jennie Jeong, Aaron Scott, Ranjana Ambannavar, Christina Zheng, Raul Mena, Jose Esteban, James Stephans, John Morlan, Joffre Baker

**Affiliations:** 1 Genomic Health Inc., Redwood City, California, United States of America; 2 Providence St. Joseph Medical Center, Burbank, California, United States of America; The Institute of Cancer Research, London, United Kingdom

## Abstract

RNA biomarkers discovered by RT-PCR-based gene expression profiling of archival formalin-fixed paraffin-embedded (FFPE) tissue form the basis for widely used clinical diagnostic tests; however, RT-PCR is practically constrained in the number of transcripts that can be interrogated. We have developed and optimized RNA-Seq library chemistry as well as bioinformatics and biostatistical methods for whole transcriptome profiling from FFPE tissue. The chemistry accommodates low RNA inputs and sample multiplexing. These methods both enable rediscovery of RNA biomarkers for disease recurrence risk that were previously identified by RT-PCR analysis of a cohort of 136 patients, and also identify a high percentage of recurrence risk markers that were previously discovered using DNA microarrays in a separate cohort of patients, evidence that this RNA-Seq technology has sufficient precision and sensitivity for biomarker discovery. More than two thousand RNAs are strongly associated with breast cancer recurrence risk in the 136 patient cohort (FDR <10%). Many of these are intronic RNAs for which corresponding exons are not also associated with disease recurrence. A number of the RNAs associated with recurrence risk belong to novel RNA networks. It will be important to test the validity of these novel associations in whole transcriptome RNA-Seq screens of other breast cancer cohorts.

## Introduction

Recent major advances in DNA sequencing, “next generation sequencing (NGS)”, provide massively parallel throughput, and data volumes that eclipse the nucleic acid information content possible with other technologies, making feasible unprecedented extensive genome analyses of groups of individuals, including analyses of sequence differences, polymorphisms, mutations, copy number variations, epigenetic variations and transcript abundance (RNA-Seq) [1]–[3]. Biomarker discovery is an attractive potential application of this new technology.

Application of older technologies, such as DNA microarray and RT-PCR platforms, have demonstrated that levels of RNA transcripts (“gene expression profiles”) stratify patients and predict outcomes in a variety of diseases, providing the basis for several important clinical tests [4]–[7]. An example is a 21-gene RT-PCR-based test, which interrogates tumor RNA to predict recurrence risk and magnitude of chemotherapy benefit in early estrogen receptor positive (ER+) breast cancer [4], [8]–[10]. This test is now used to guide treatment decisions for about half of ER+ breast cancer patients in the U.S. [11].

The NGS methods described here enable transcriptome-wide cancer biomarker discovery with archival fixed paraffin-embedded (FFPE) tissue. Many thousands of FFPE tissue blocks associated with mature clinical records exist in hospital pathology archives. These can be used for tumor gene expression profiling and therefore enable rapid clinical biomarker discovery in studies that are statistically well-powered [12]–[15]. The use of FFPE tissue in commercial clinical laboratory tests also aligns with the standard clinical practice of creating FFPE tissue specimens from biopsies and surgical resections. Because patient FFPE tissue biopsy material frequently contains limited amounts of tissue, we sought to develop an NGS RNA-Seq method that is compatible with low input levels of archival FFPE RNA.

We have carried out RNA-Seq analysis on FFPE tumor RNA from a cohort of 136 breast cancer patients with tumor tissue at the time of resection and clinical outcomes for disease recurrence. This tumor RNA was originally screened by RT-PCR in the biomarker discovery phase of the development of the 21 gene breast cancer recurrence risk assay [4], [16]. RNA-Seq analysis of these tumors provides opportunities both to determine whether biomarker RNAs originally discovered by RT-PCR could be rediscovered by RNA-Seq, and to identify potential new RNA risk markers.

Although most current biological knowledge centers on the fraction of the genome that encodes proteins, most mammalian transcripts are non-protein-coding intronic and intergenic sequences [17], [18], and important biological functions of RNAs within both of these classes are now widely recognized. For example, microRNAs, which number over 1,000, and are commonly encoded in introns, regulate mRNA transcription and stability [19], [20]. Large intergenic non-coding RNAs (lincRNAs), which number in the thousands, regulate gene transcription [21]–[23]. This study suggests associations between hundreds of both coding and non-coding RNAs and breast cancer recurrence risk.

## Results

Patient clinical characteristics are given in Table S1. One-hundred and ten patients (81%) had no involved nodes. There was a mixture of chemotherapy and hormonal therapy usage. Estrogen receptor (ER) status was not included in patient records. We therefore used normalized estrogen receptor gene (ESR1) mRNA levels obtained in the present RNA-Seq study to identify 111 tumors as estrogen-receptor positive and 25 as estrogen-receptor negative (Figure S1). Use of RT-PCR rather than RNA-Seq for this purpose yielded similar but not identical results, identifying as ER+ two more patients, for a total of 113. Archive ages of FFPE tumor blocks ranged from 5 to 12.4 years (median 8.5 years).

RNA-Seq results were successfully generated for all 136 patients, with an average of 43 million median reads per patient (86 million median reads per Illumina Hiseq 2000 flow cell lane). Sixty-nine percent of these uniquely mapped to the human genome: 19.2% to exons, 64.9% to introns, and 15.9% to intergenic regions. Ribosomal RNA accounted for less than 0.3% of the total reads. On average, 17,248 Refseq transcripts were detected per patient, 66% with greater than 10 counts, and 47% with greater than 100 counts (Table S2).

### Evaluation of Whole Transcriptome RNA-Seq as a Platform for Biomarker Discovery

**Figure 1:**
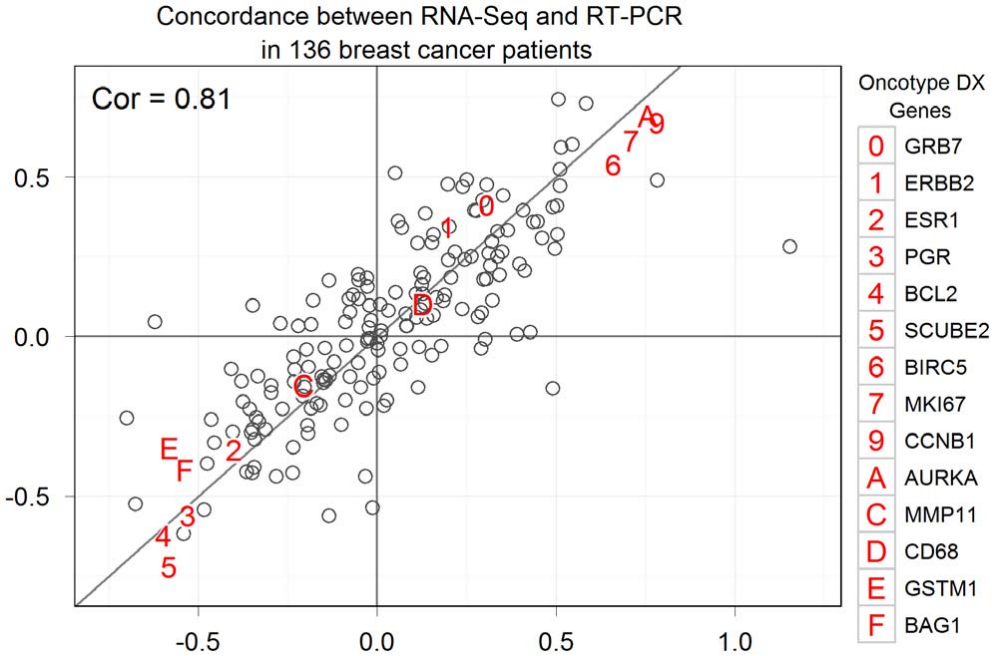
Scatter plot of recurrence risk hazard ratios of RNA sequences. RT-PCR results versus RNA-Seq results. Each point represents a distinct RNA. Genes in the 21-gene breast cancer recurrence risk assay are marked with alphanumeric symbols.

The spectrum of log base_2_ hazard ratios for each of the 185 genes screened in the historical RT-PCR-based study is shown in Figure 1 (on x-axis). Fourteen of the sixteen cancer-related mRNAs in the 21-gene breast cancer test were assayed in that study (shown by highlighted alpha-numeric symbols in Figure 1). Significantly, Figure 1 shows that hazard ratios obtained based on RNA-Seq are highly concordant with those obtained by RT-PCR [24] (Lin concordance correlation: 0.810; Pearson correlation coefficient: 0.813).

**Figure 2:**
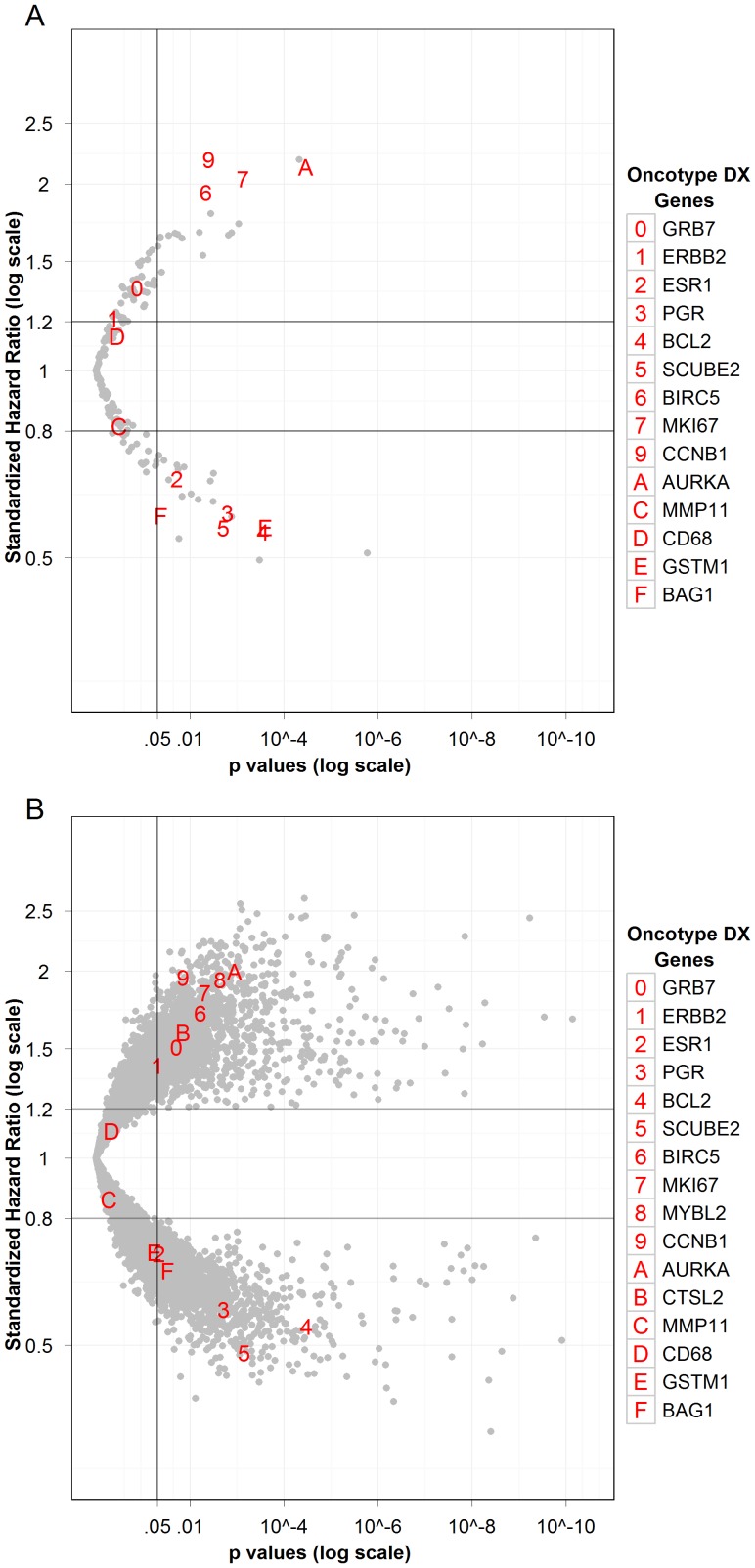
Relationship of increased RNA expression to risk of breast cancer recurrence in 136 breast cancer patients. Each point represents a distinct RNA. The magnitude of the effect size is given by the hazard ratio from Cox proportional hazard analysis and statistical significance by P-Value. Genes in the 21-gene breast cancer recurrence risk assay are marked with alphanumeric symbols. **A.** Analysis of 192 genes measured by RT-PCR. **B.** Analysis of assembled RefSeq transcripts as measured by whole transcriptome RNA-Seq.

**Figure 3:**
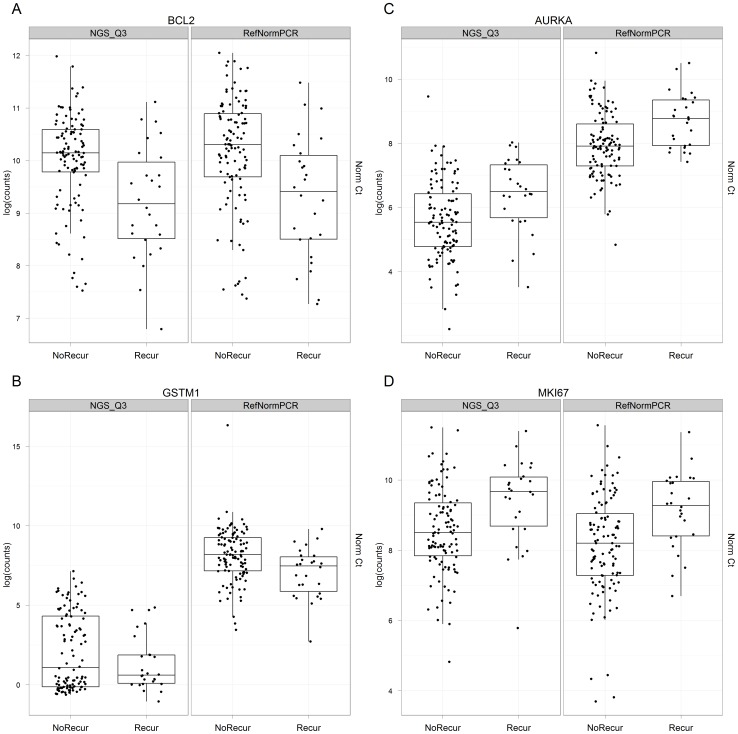
Box plots of normalized expression values of RNAs in breast cancer patients, stratified by recurrence status. Each point represents a patient tumor. The bottom and top of the box are the 25^th^ and 75^th^ percentiles and the band within the box is the 50^th^ percentile (the median) of the points in the group. The ends of the vertical lines represent the lowest datum still within 1.5 interquartile range of the lower quartile, and the highest datum still within 1.5 interquartile range of the upper quartile. Values from RNA-Seq (left panel) and RT-PCR (right panel) are shown: **A.** BCL2. **B.** GSTM1. **C.** AURKA. **D.** MKI67.

Figure 2A also displays results from the historical [4], [16] RT-PCR 185 candidate gene screen of the Providence 136 patient cohort, relating increasing mRNA expression to recurrence risk hazard ratios and statistical significance. As shown, 14 of the 16 cancer-related genes in the 21-gene breast cancer test panel [4] were identified with Hazard Ratios greater than 1.2 or less than 0.8 and P values <0.05. Both the effect sizes and statistical significance of these 14 genes were similar when screening was carried out by whole transcriptome RNA-Seq rather than RT-PCR (compare Figures 2A and 2B). This is shown in detail on a gene-by-gene basis in box plots (Figure 3 and Figure S2, A-I). (It is noted that the distribution of GSTM1 counts is bimodal in the RNA-Seq data, with about half of the samples registering no counts. This is consistent with evidence that this gene is deleted in about half of Caucasians [25], [26]. The RT-PCR GSTM1 assay does not indicate this loss, presumably because this assay detects multiple members of the GSTM1 family [27].)

Significantly, RNA-Seq further associates many transcripts annotated in RefSeq with disease recurrence: a total of 1307 at FDR<10% (Table S3). These are hereafter referred to as identified RefSeq RNAs. In contrast, the 192 gene RT-PCR study identified 32 RNAs at FDR<10%, and consumed five-fold more input RNA. Together, these results indicate that RNA-Seq can provide a practical, sensitive and precise platform for genome-wide biomarker discovery in FFPE tissue.

To evaluate the impact of transcript abundance on initial biomarker discovery the 1307 Identified RefSeq RNAs were binned with respect to count abundance (Table 1). About 30% of these transcripts are present at less than 10 median counts. The percent of RNAs identified decreases but is not dramatically different as median counts decrease from greater than 1,000 to 10–99. Even at median counts less than 10, the percent of RNAs identified fell by less than half compared to sequences present at higher abundance.

**Table 1:** Relation of median RNA count with frequency of RNA identification at FDR<10%.

Median RNA Count	Number of RNAs*	Number of RNAs Identified atFDR<10%	Percent of RNAs Identified at FDR<10%
<10	5817	286	4.9%
10–99	6245	399	6.4%
100–999	7657	551	7.2%
≥1000	743	71	9.6%
Total	20,462	1307	6.4%

*Number across entire 136 patient population.

The performance of the RNA-Seq technology and resulting identified RefSeq RNAs was further evaluated using public gene expression data from an independent cohort of breast cancer patient tumors that had been assayed by DNA microarray technology. The microarray data set was assembled by merging patient sets published in two articles [28], [29], providing data on 337 patients (“NKI dataset”). Among the 11,659 genes common to both platforms, there is highly significant agreement in the classification of genes as prognostic (Table S4), but concordance falls off as transcript abundance decreases. For identified RNA-Seq RNAs present at >100 counts, 44% are significant in the NKI dataset, but at the lowest quartile of RNA-Seq count abundance, the level of agreement is not statistically significant (Table S4).

### RefSeq Transcripts and Gene Networks that Associate with Risk of Breast Recurrence

Among the 1307 identified RefSeq RNAs, many relate to recurrence with very high statistical significance (Table S3). Estimated standardized hazard ratios corrected for regression to the mean are as high as 1.66. Uncorrected hazard ratios range from approximately 0.4 to 2.5. The ratio of RNAs for which high expression associates with increased risk of cancer recurrence, versus decreased risk is approximately 1.

**Figure 4:**
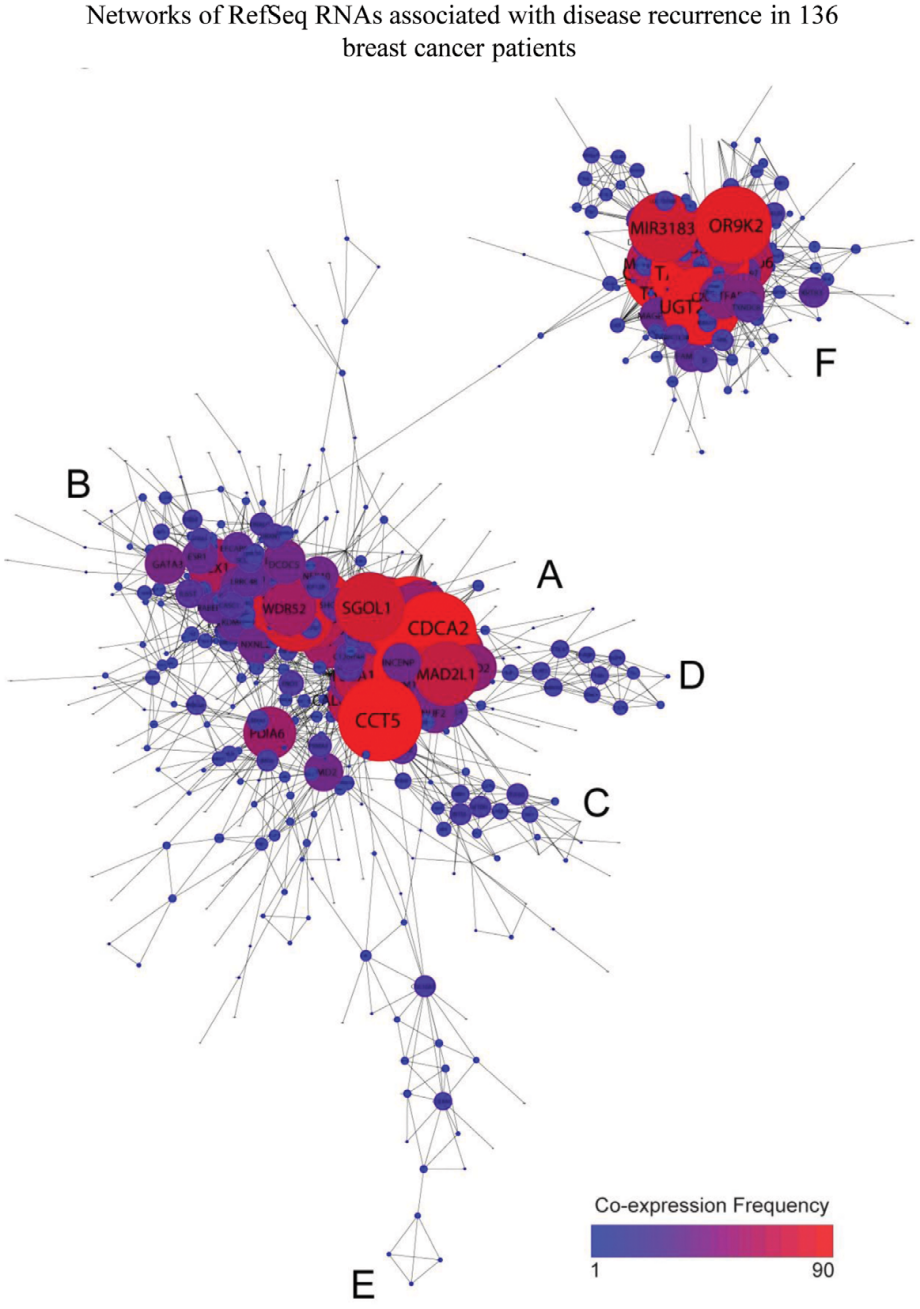
Multiple RefSeq RNA networks with common biological themes. Among the set of 1307 identified RefSeq RNAs, a subset was selected that contains all RNAs that co-express (at R>0.6) with at least one other RNA in the set of 1307. Cytoscape 2.8 visualization [31], [32]. The degree of node interaction (total number of co-expression interactions) is mapped to node size and color (indicated by scale). Biological annotation of genes that are highly represented in identified networks is indicated by letter labels. **A**: cell cycle; **B**: co-expression with ESR1; **C**: genes mapping to Ch17q23-24; **D**: genes mapping to Chr821-24; **E**: genes mapping to Chr9q22; **F**: olfactory signaling, glucose metabolism, glucuronidation.

Hierarchical clustering [30] of the 1307 identified RefSeq RNAs (Figure S3) suggests the presence of co-expressed gene networks. Cytoscape [31], [32] was used to evaluate that subset of these RNAs for which each RNA member correlates in its expression with at least one other RNA at R ≥0.6. Figure 4 graphically represents the resulting correlation matrix of 597 RNAs and 4011 interactions. [31], [32]. One prominent (51 member) RefSeq RNA network is enriched in RNAs with Reactome database [33] annotations that are functionally related to regulation of the cell cycle and mitosis, and associates with poor prognosis. A second network is enriched in RNAs that co-express with the estrogen receptor gene (ESR1) and associate with reduced recurrence risk. [4], [9]. The expression of ESR1 itself is not statistically associated with disease outcome in our RNA-Seq results, nor was it previously found to be significant in this cohort by RT-PCR analysis (Figure 2A).

This analysis also reveals several novel RNA networks, three of which map to discrete cytogenetic bands (Figure 4): a network of twelve RNAs mapping to a 6.6 megabase region of Chr17q23-24; a fourteen RNA network mapping to a 47 megabase span on Chr8q21-24; a network of five poor prognosis RNAs mapping to a 289 kilobase region located at Chr9q22 (not labeled in Figure 4); finally, a large (134 member) RNA network that has strong Gene Ontology and Biocarta annotations to olfactory signaling, glucose metabolism, and glucuronidation. Nine of the transcripts in this latter network encode olfactory receptors. Fourteen are microRNA precursors. Most of the RNAs in this network are rare (raw median counts less than 10). All but two associate with poor prognosis.

ER status, which is often described in clinical practice in binary terms as ER+ and ER- via immunohistochemistry evaluation of breast tumors, dichotomizes breast cancer with respect to clinical outcome and gene expression profiles [34]–[36]. While ER status was not part of patient records for this study cohort, we used RNA-Seq ESR1 counts to separate patients (Figure S1). This analysis is presented in Table S5, acknowledging various factors that compromise its clinical reliability: the novel method of defining ER status; limited statistical power because of small population size (10 recurrence events); the absence of hormonal therapy in a significant fraction of those patients that we defined as ER+. Administration of hormonal therapy (e.g., tamoxifen or an aromatase inhibitor) is current standard clinical practice, and both significantly decreases recurrence risk [37] and influences the nature of biomarkers that predict recurrence [38], [39]. Nevertheless, this analysis does identify the expected cell cycle gene signature as a marker of high recurrence risk (exemplified by the genes CCNA2, CENPN, KIF20, ARPP19 and BUB3). In all, expression of 363 RefSeq transcripts relate to recurrence risk at FDR<10% (Table S5). Within this set of transcripts the most prominent RefSeq RNA network observed using Cytoscape as described above, is similar to the rare transcript network that was identified in analysis of the entire 136 patient cohort. All RNAs in this network associate with increased risk of disease recurrence.

### Analysis of Intronic and Intergenic Sequences

Reads mapping to intronic regions of the genome account for ∼65% of all of the sequence data. Introns tend to co-express with exons of the same genes (median R = 0.67), although these correlations vary over a wide range, from roughly zero to over 0.9. A large number (1698) of intronic RNAs associate with breast cancer recurrence (at FDR <10%; non-directional analysis; Table S6), with ranges of hazard ratios and p-values that are similar to those of the above-identified 1307 RefSeq RNAs.

For two thirds (1154) of the identified intronic transcripts, the corresponding assembled exons are not also discovered, as indicated by comparing Tables S3 and S6. Among the 100 most statistically significant intronic RNAs this fraction is 0.44. Genes for which intronic but not exonic RNAs are discovered might simply be the result of technical signal-to-noise ratios favoring discovery of introns, because average counts for introns are more than threefold higher than for exons. However, we find that in the population of exons that are not discovered along with discovered cognate introns, average exon abundance is just modestly lower than in the entire population of discovered exons (average counts 244 versus 312, respectively), consistent with the possibility that introns frequently carry qualitatively novel prognostic information.

We used two approaches to search for biomarkers within the population of intergenic RNAs, first by interrogating reads that map to 2,500 well-documented lincRNAs [21]. Twenty-two of these (Table S7), associate with breast cancer recurrence risk at FDR<10%. Second, intergenic transcripts were screened more broadly using a computational algorithm (Method S1) to identify clusters of reads that map to intergenic regions of the genome in one or more of the tumor specimens. The number of reads mapped to these clusters was used as a measure of the expression of putative intergenic transcripts. Altogether 2101 such transcripts were identified, 775 of which are contained in or overlap with lincRNAs that have been identified previously in one or more studies of non-coding transcripts [21], [22], [40]–[45]. Expression of 194 (9%) of these putative intergenic transcripts correlates with disease recurrence in the 136 patient cohort, at FDR <10%. This list of 194 was further condensed by merging clusters of reads separated by <1000 bp to produce a set of 69 putative intergenic transcripts associated with recurrence of breast cancer (Table S8). This merging of clusters is supported by the observation that the median correlation coefficient for co-expression of the merged clusters is extraordinarily high (median R = 0.94). Non-merged transcripts exhibit weak co-expression (median R = 0.27).

## Discussion

The present report is the result of our development of RNA-Seq methods suitable for biomarker discovery in fixed clinical tissue. The library chemistry described accommodates low amounts of archival FFPE tissue RNA, preserves strand-of-origin information, is compatible with sample indexing, and quantifies transcripts with sufficient sensitivity and precision for biomarker discovery in valuable clinical tissue specimens. Data analysis methods were selected from a number of tested options. Results generated using 5–12 year old FFPE tumor tissue from 136 breast cancer patients, are concordant with RT-PCR data. Study results also indicate that effective biomarker identification is possible with multiplexed samples.

RNAs that are new putative markers of breast cancer recurrence risk are identified here, which, singly and in sets, frame hypotheses to test in later studies of other breast cancer patient cohorts. While we associated ∼1300 RefSeq RNAs (which are mostly mRNAs) with breast cancer prognosis (at FDR<10%), more than half of the total RNAs identified as prognostic lie in the ∼98% of the genome that does not code for proteins. It is noteworthy that, for most of the intronic RNAs identified as prognostic, their cognate assembled exons were not also identified as prognostic, consistent with the possibility that these intronic sequences carry biomarker information not captured in gene coding sequence. Most of the identified non-coding RNAs are very long sequences that have low counts per kilobase, and the power for identifying longer sequences is expected to be higher because of the increased counts. However, each evaluated RNA effect size (the hazard ratio for its association with recurrence) is effectively estimated by comparisons of sequence expression among patients. To the extent that shorter RNAs are handicapped in signal strength, they are handicapped equally within an RNA species, so they do not bias the analysis of each individual sequence. Future studies of other breast cancer cohorts will reveal whether rare transcripts identified here prove to be robust biomarkers.

To analyze intergenic transcripts we evaluated a set of lincRNAs described in the recent literature [21] and also transcripts identified by a new algorithm that interrogates an entire population of transcriptomes to identify intergenic transcripts based on transcript abundance and density. Development of biostatistical and bioinformatic programs and databases for NGS data analysis is a very active area [45]–[48]. Subsequent analysis by new biostatistical and bioinformatics methods will further test and validate study conclusions.

The 1307 RefSeq RNAs associated with prognosis were also examined for prognostic significance in tumors from an independent cohort of patients in which DNA microarray technology was used to profile gene expression (public NKI data set) [28], [29]. About half of these 1307 transcripts could be found as features on the microarrays. Of these shared RefSeq transcripts, about 40% were found to be prognostic in the NKI data set (P<10^−16^). There is no significant inter-study concordance for the lower abundance quartile of Ref-Seq transcripts, plausibly attributable to the fact that signal-to- noise ratios in both technologies decrease as transcript numbers decrease.

A number of DNA microarray and RT-PCR studies of early breast cancer have identified as markers of poor prognosis a network of co-expressed mRNAs that regulate the cell cycle [4], [49]. The original RT-PCR interrogation of this 136 patient cohort identified a number of these transcripts and this co-expressed network strongly emerges in the RNA-Seq analysis of this cohort. A network of genes that co-express with ESR1, the estrogen receptor gene, has also been linked to decreased breast cancer recurrence risk in several published studies [4], [9] and also emerges in our RNA-Seq results. Several novel networks of RefSeq transcripts also track with increased breast cancer recurrence risk. The largest of these (containing 134 RNAs) is heavily populated with low abundance RNAs, and includes a number of pre-microRNAs and olfactory receptor mRNAs. It may mark decreased stringency of certain transcriptional controls.

We have not analyzed these RNA-Seq data for either mutations or alternatively spliced isoforms. While we plan to do these evaluations, the laboratory protocols used here are not optimal for these assessments. The chemistry of our libraries is compatible with paired-end sequencing (not performed in the present study), which is highly desirable for analysis of splice variants and gene fusions [50]. Given its fragmented condition, FFPE tissue RNA could present a formidable challenge to assembly of differentially spliced isoforms. We do anticipate that the library preparation methods described here will yield high quality RNA-Seq data from non-fixed fresh or frozen tissue, based on unpublished preliminary data.

In conclusion, this work describes the application of RNA-Seq methods with sufficient sensitivity and reproducibility to enable biomarker discovery in archival FFPE tissue. Whole transcriptome RNA-Seq reveals hundreds of new coding and non-coding transcripts, as well as heretofore unappreciated gene networks that strongly associate with breast cancer recurrence in this study cohort. Recognizing the challenges for development of robust gene signatures associated with clinical variables [51], the transcripts identified here should be explored in future screens for biomarkers of breast cancer recurrence risk.

## Materials and Methods

One hundred thirty-six primary breast cancer FFPE tumor specimens with clinical outcomes were provided by Providence St. Joseph Medical Center (Burbank, CA), with institutional review board approval [4], [16]. The time to first recurrence of breast cancer or death due to breast cancer (including death due to unknown cause) was determined from these records. Patients who were still alive without breast cancer recurrence or who died due to known other causes were considered censored at the time of last follow-up or death. These tumor specimens were used for biomarker discovery in the development of the 21-gene breast cancer recurrence risk assay [4], [16]. For the present study, all 136 specimens had adequate RNA remaining. Among the 136 patients, 26 experienced breast cancer recurrence or death due to breast cancer or unknown causes.

### RNA-Seq Sample Preparation and Sequencing

Total RNA was prepared from three 10-µm-thick sections of FFPE tumor tissue as previously described using the MasterPure™ Purification Kit (Epicentre® Biotechnologies, Madison, WI) [15], [16]. One hundred nanograms of the isolated RNA were depleted of ribosomal RNA as described (Morlan et al. manuscript submitted for publication). Sequencing libraries for whole transcriptome analysis were prepared using ScriptSeq™ mRNA-Seq Library Preparation Kits (Epicentre® Biotechnologies, Madison, WI). To increase library yield, additional incubation for 90 minutes at 37°C was carried out in the cDNA synthesis step prior to addition of Finishing Solution 1. The presence of fresh DTT (dithiothreitol) in the reaction buffer is critical for optimal cDNA synthesis using archival FPET RNA and this method. After 3′-terminal tagging, the di-tagged cDNA was purified using MinElute® PCR Purification Kits (Qiagen®, Valencia, CA). Two 6-base index sequences were used to prepare bar-coded libraries for sample multiplexing (RNA-Seq barcode primers; Epicentre® Biotechnologies, Madison, WI). PCR was carried out through 16 cycles to generate the second strand of cDNA, incorporate barcodes, and amplify libraries. The amplified libraries were size-selected by a solid phase reversible immobilization, paramagnetic bead-based process (Agencourt® AMPure® XP System; Beckman Coulter Genomics, Danvers, MA). Libraries were quantified by PicoGreen® assay (Life Technologies, Carlsbad, CA) and visualized with an Agilent Bioanalyzer using a DNA 1000 kit (Agilent Technologies, Waldbronn, Germany).

TruSeq™ SR Cluster Kits v2 (Illumina Inc.; San Diego, CA) were used for cluster generation in an Illumina cBOT™ instrument following the manufacturer’s protocol. Two indexed libraries were loaded into each lane of flow cells. Sequencing was performed on an Illumina HiSeq®2000 instrument (Illumina, Inc.) by the manufacturer’s protocol. Multiplexed single-read runs were carried out with a total of 57 cycles per run (including 7 cycles for the index sequences).

### Data Quality Assessment

Each sequencing lane was duplexed with two patient sample libraries using a 6-base barcode to differentiate between them. The mean read ratio +/−SD between the two samples in each lane was 1.05±0.38 and the mean +/−SD percentage of un-discerned barcodes was 2.08%±1.63%. Using principal components analysis and other exploratory data analysis methods, we found no systematic differences among samples associated with flow cell or barcode.

In a run-in phase of the study, we prepared duplicate libraries for 8 samples selected at random from the study set of 136. RefSeq RNA coverage for these libraries ranged between 3.1M and 6.7M uniquely mapped reads. Log count Pearson correlations among duplicate libraries ranged between 0.947 and 0.985. Single libraries were prepared for the remaining 128 samples and distributed in duplex mode among the lanes of 8 flow-cells. Sequencing in 3 lanes failed. Two libraries had low yield, resulting in low coverage. Three lanes were flagged by various Illumina process monitoring indices. New libraries for samples that had low yield were prepared and sequenced. Libraries in the failed and flagged lanes, as well as some of the low coverage samples were re-sequenced. Replicate correlations among all sequenced samples were very high, 0.985 for the samples with the high cluster density in the original run, and over 0.990 for all others. For data analysis, we used the data for one of each of the duplicate libraries from the run-in experiment. In cases in which new libraries were prepared, and for the samples in the failed and flagged lanes, we used the reads from the subsequent run. For the samples with low coverage for which we reprocessed the library, reads from the two runs were pooled. For the rest of the samples, we used the reads from the single lane. Results differed little when other data analysis procedures were used, for example, using only the second run when libraries were reprocessed.

### Statistics and Bioinformatics

With the exception noted below, all primary analysis of sequence data was performed in CASAVA 1.7, the standard data processing package from Illumina. De-multiplexing of sample indices was set with 1 mismatch tolerance to separate the two samples within each lane. Raw FASTQ sequences were trimmed (6 bases from the 5′side and 8 bases from the 3′side) before mapping to the human genome (UCSC release, version 19), to address 3′ end adapter contamination, random RT primer artifacts, and 5′ end terminal-tagging oligonucleotide artifacts. Mapping started from a single seed of 32 base pairs with two mismatches allowed; gap penalties were allowed using ELAND2 provided by Illumina. The libraries as prepared contain strand-of-origin (directional) sequence information. Annotated RNA counts (defined by refFlat.txt from UCSC) were calculated by CASAVA 1.7 both with and without consideration of strand-of-origin information. Although retained in the mapping process, CASAVA does not provide directional counts by default. These counts were obtained by splitting the mapped (export.txt) file into two parts, one with sense strand counts, the other with antisense strand counts, and processing them independently. Raw FASTQ sequence was mapped with Bowtie [52] in parallel with CASAVA to count ribosomal RNA transcripts.

Data were analyzed in 3 categories: first, RefSeq RNAs, about 80% of which are exon sequences, consolidated for each gene; second, intronic RNA sequences, consolidated for each gene; third, intergenic sequences, operationally defined as non-RefSeq, non-intronic sequences (Data for this study have been deposited in the Dryad Repository: http://dx.doi.org/10.5061/dryad.q760h). RNAs for which none of the 136 specimens yielded 5 or more counts were excluded from analysis. Of 21,283 total RefSeq transcripts counted by CASAVA, 821 had a maximum count less than 5, leaving 20,462 RefSeq transcripts for analysis. Similar to a recently published procedure described by Bullard *et al.* [48] log_2_ raw RNA counts (setting the log_2_ for a 0 count to 0) were normalized by subtracting the 3^rd^ quartile of the log_2_ RefSeq RNA counts and adding the cohort mean 3^rd^ quartile (“3^rd^ quartile normalization”). For normalization of RefSeq and intergenic RNA data, RefSeq transcript data were used. For normalization of intronic RNA data, intronic transcript data were used. Use of third quartile normalization effectively mitigated trends in overall coverage related to sample age and produced stable estimates of expression with relative log expression (RLE, individual gene log_2_ count minus within-patient median log_2_ count) values that were centered on zero and relatively tightly distributed around 0, an indicator of effective normalization.

Standardized hazard ratios for breast cancer recurrence for each RNA, that is, the proportional change in the hazard with a 1-standard deviation increase in the normalized expression of the RNA, were calculated using univariate Cox proportional hazard regression analyses [53]. The robust standard error estimate of Lin and Wei [54] was used to accommodate possible departures from the assumptions of Cox regression, including nonlinearity of the relationship of gene expression with log hazard and non-proportional hazards. False discovery rates (FDR, *q*-values) were assessed using the method of Storey [55] with a “tuning parameter” of λ = 0.5. Analyses were conducted to identify true discovery degree of association (TDRDA) sets of RNAs with absolute standardized hazard ratio greater than a specified lower bound while controlling the FDR at 10% [56]. Taking individual RNAs identified at this FDR, the analysis finds the maximum lower bound for which the RNA is included in a TDRDA set. Also computed was an estimate of each RNA’s actual standardized hazard ratio corrected for regression to the mean [56].

Expression of 192 transcripts in the same tumor RNAs was measured using previously described RT-PCR methods [15]. Standardized hazard ratios associating the expression of each gene (normalized by subtracting each gene’s crossing threshold (C_T_) from the cohort median C_T_) with cancer recurrence were computed using the same methods used for evaluation of the RNA-Seq data.

Intergenic regions were identified by a novel program that evaluates intergenic regions having wide variations in length, and uses data from a population of subjects rather than an individual subject. Briefly, overlapping reads from all 136 patients were combined based on their human genome mapping coordinates, creating clusters of individual islands. Nearby islands were grouped by a merging tolerance criterion into regions of interest. Putative novel intergenic transcripts were then defined by filtering out transcripts with known refFlat.txt annotations.

## Supporting Information

Figure S1 **Identification of ESR1-positive patients by RNA-Seq analysis.** Normalized values of ESR1 and PGR in 136 breast cancer patients are represented in a scatter plot. Each symbol represents a different patient. Because in human breast cancer it is rare for a tumor to be both PGR positive and ER negative, or to be PGR negative and ER positive, the distribution of both PGR and ESR1 data were used to set cutoffs for calling patient ESR1 status. The vertical and horizontal cutoffs were set by visual inspection of the data.
(TIFF)

Figure S2 **Box plots of normalized expression values of RNAs in breast cancer patients, stratified by recurrence status.** Each point represents a patient tumor. The bottom and top of the box are the 25^th^ and 75^th^ percentiles and the horizontal band within the box is the 50^th^ percentile (median) of the points in the group. The ends of the vertical lines represent the lowest datum still within 1.5 inter-quartile range of the lower quartile, and the highest datum still within 1.5 inter-quartile range of the upper quartile. Values from RNA-Seq (left panel) and RT-PCR (right panel) are shown. A. BAG1; B. BIRC5; C. CCNB1; D. CD68; E. ESR1; F. ERBB2; G. GRB7; H. MMP11; I. PGR.
(PDF)

Figure S3 **Heat map for expression of Identified RefSeq RNAs in 136 breast cancer patients.** All 1307 Normalized expression values of RefSeq RNAs that were found to be associated with risk of breast cancer recurrence (Table S3) are represented on the vertical axis. Patients are represented on the horizontal axis at the top of the figure.
(TIFF)

Table S1 **Patient case characteristics and outcomes.**
(TIFF)

Table S2 **Providence RNA-Seq 136 patient run report.**
(TIFF)

Table S3 **Assembled RefSeq RNAs Identified as Associated with Risk of Breast Cancer Recurrence in 136 Breast Cancer Patients.**
(XLSX)

Table S4 **Agreement between genes identified using public microarray data and the NGS data.**
(TIFF)

Table S5 **Assembled RefSeq RNAs Identified as Associated with Risk of Breast Cancer Recurrence in 111 Breast Cancer Patients Designated as ESR1 Positive.**
(XLSX)

Table S6 **Assembled Intron RNAs Identified as Associated with Risk of Breast Cancer Recurrence in 136 Breast Cancer Patients.**
(XLSX)

Table S7 **LincRNAs Identified as Associated with Breast Cancer Recurrence in 136 Breast Cancer Patients.**
(TIFF)

Table S8 **Intergenic Region Locations Identified as Associated with Risk of Breast Cancer Recurrence in 136 Breast Cancer Patients.**
(XLSX)

Method S1 **The uniquely mapped reads from all (136) patients were filtered to a depth of 1 read to eliminate potential noise from mis-mapping, and other sources.** These reads were clustered into individual read islands based on the overlap of their human genome map coordinates, yielding 12,750,071 islands. Nearby islands were consolidated by a merging distance cutoff that was calculated by a maximum likelihood estimation (MLE) to maximize overlap of identified regions with known genes. This yielded a cutoff of 30 base pairs (bp) and 6,633,258 regions of interest (ROIs). These were then filtered by the three retention criteria: 1) average read count ≥5 across the tested patient population, 2) ROI length ≥100 base pairs, 3) read depth (average read number divided by the length of the ROI) ≥0.075. ROIs among the remaining 23,024 were classified as intergenic if they did not overlap with the transcripts annotated in the UCSC refFalt.txt file. A total of 2101 intergenic region ROIs were obtained from this process.
(DOCX)
