# Supplementary figures and images for: Whole Transcriptome RNA-Seq Analysis of Breast Cancer Recurrence Risk Using Formalin-Fixed Paraffin-Embedded Tumor Tissue
Source: PLoS One. 2012 Jul 13;7(7):e40092. doi: 10.1371/journal.pone.0040092 (PMC3396611; doi:10.1371/journal.pone.0040092)

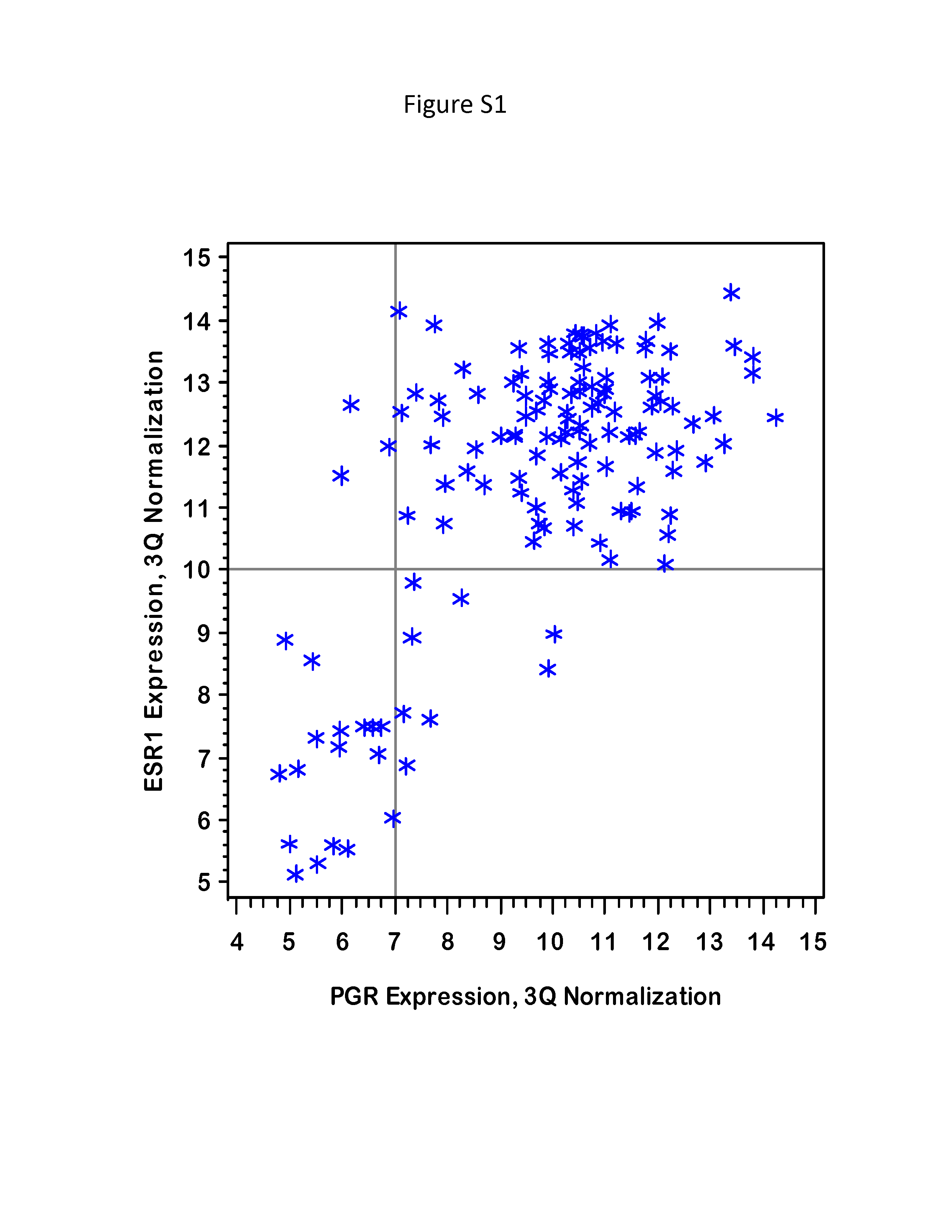

Supplement: Figure S1 — Identification of ESR1-positive patients by RNA-Seq analysis. Normalized values of ESR1 and PGR in 136 breast cancer patients are represented in a scatter plot. Each symbol represents a different patient. Because in human breast cancer it is rare for a tumor to be both PGR positive and ER negative, or to be PGR negative and ER positive, the distribution of both PGR and ESR1 data were used to set cutoffs for calling patient ESR1 status. The vertical and horizontal cutoffs were set by visual inspection of the data. (TIFF) [file pone.0040092.s001.tiff]

Figure S2

2A

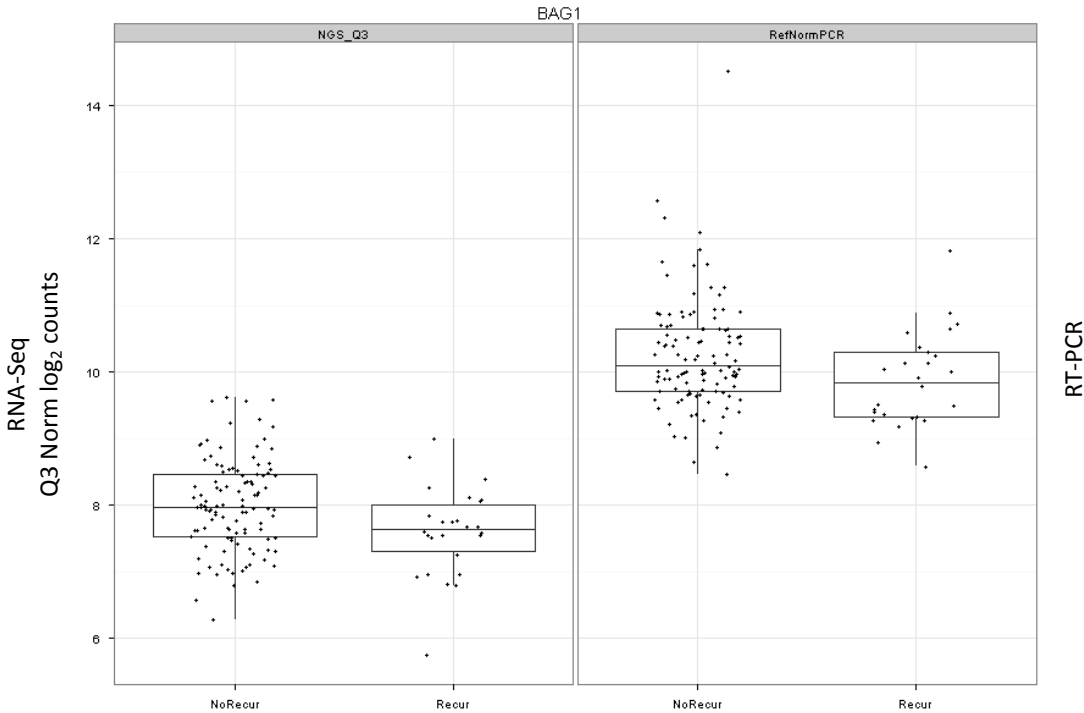

2B

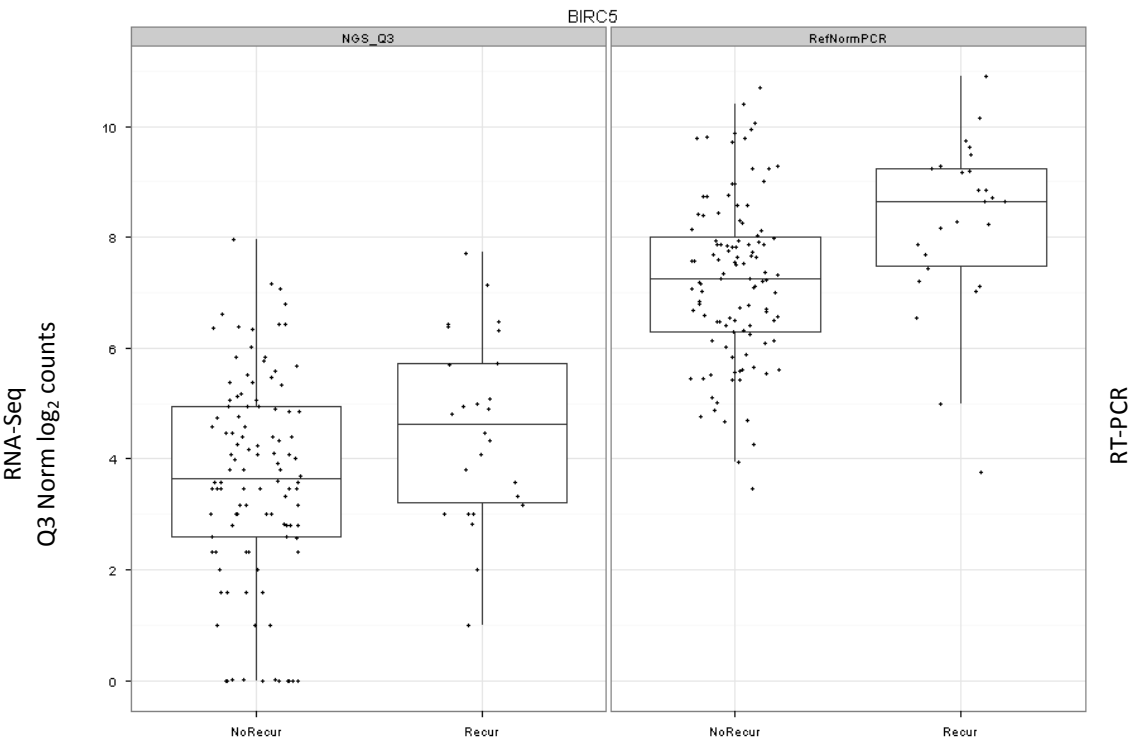

2C

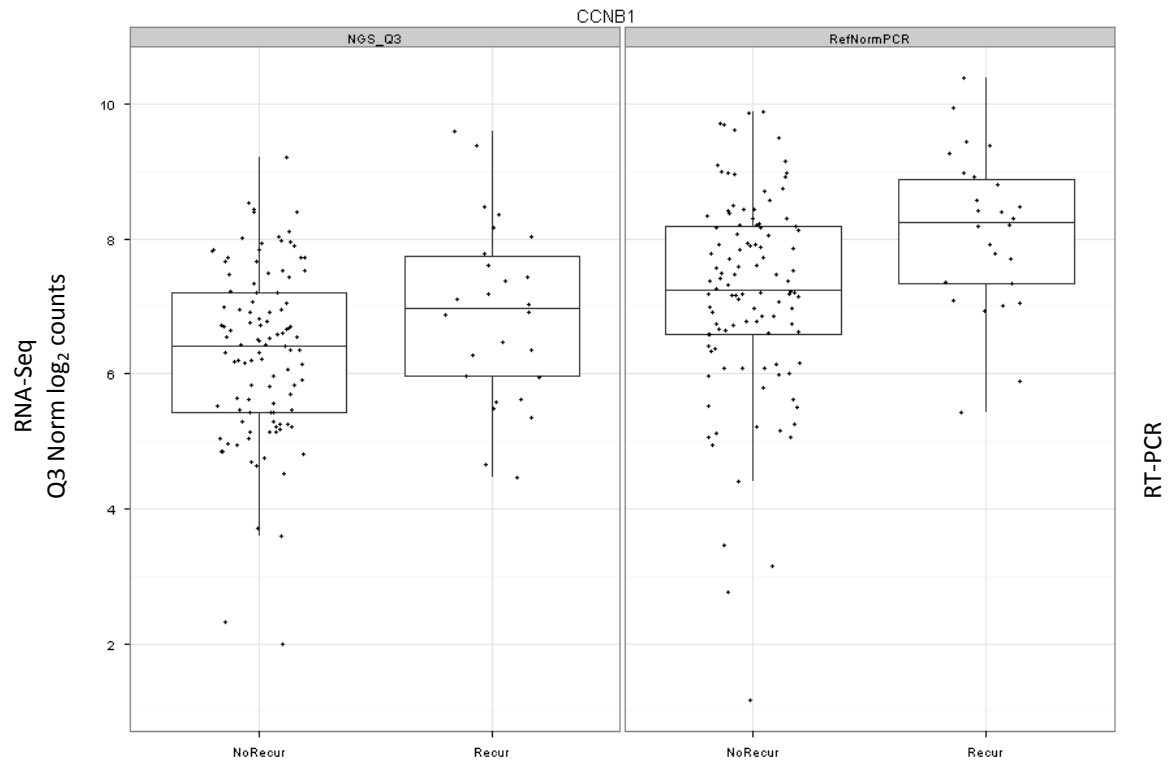

2D

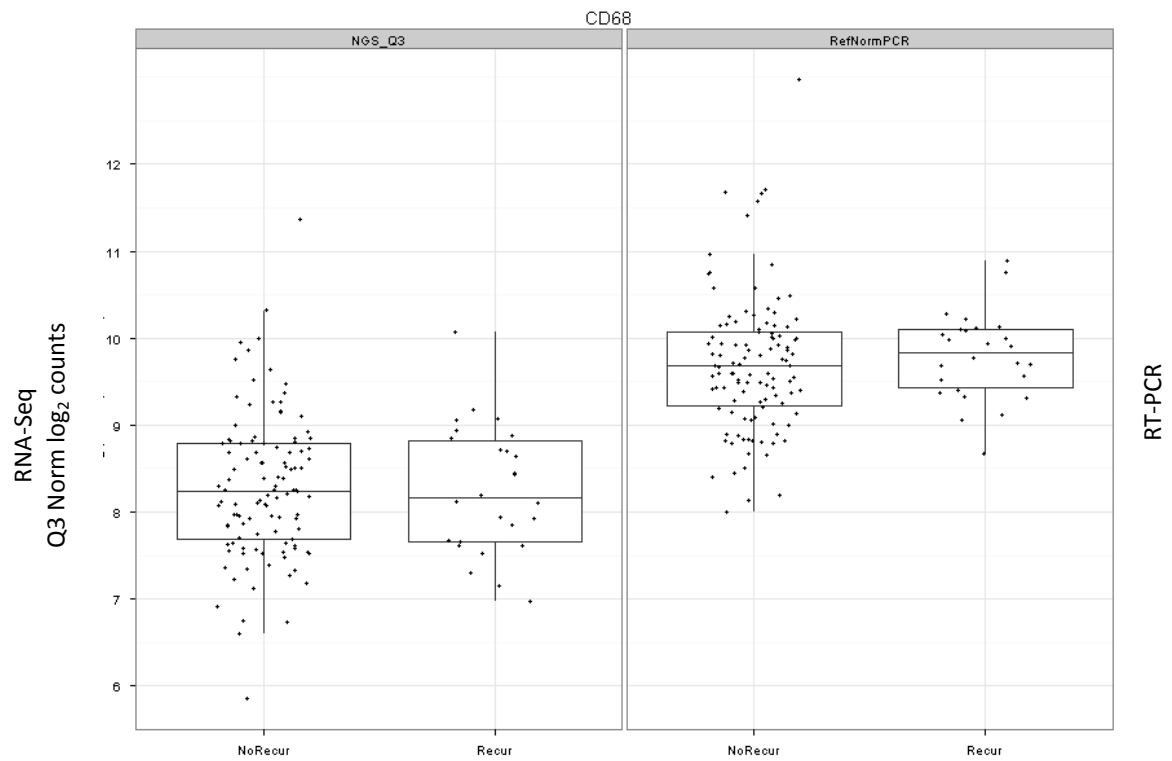

2E

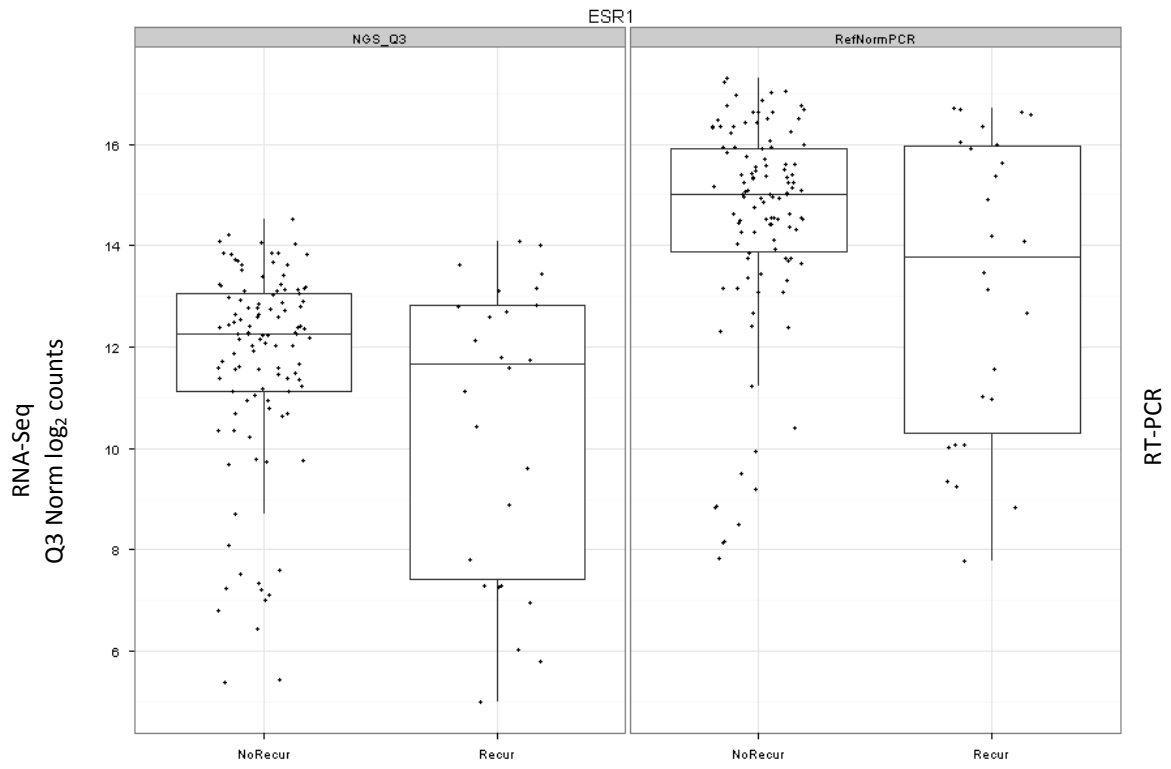

2F

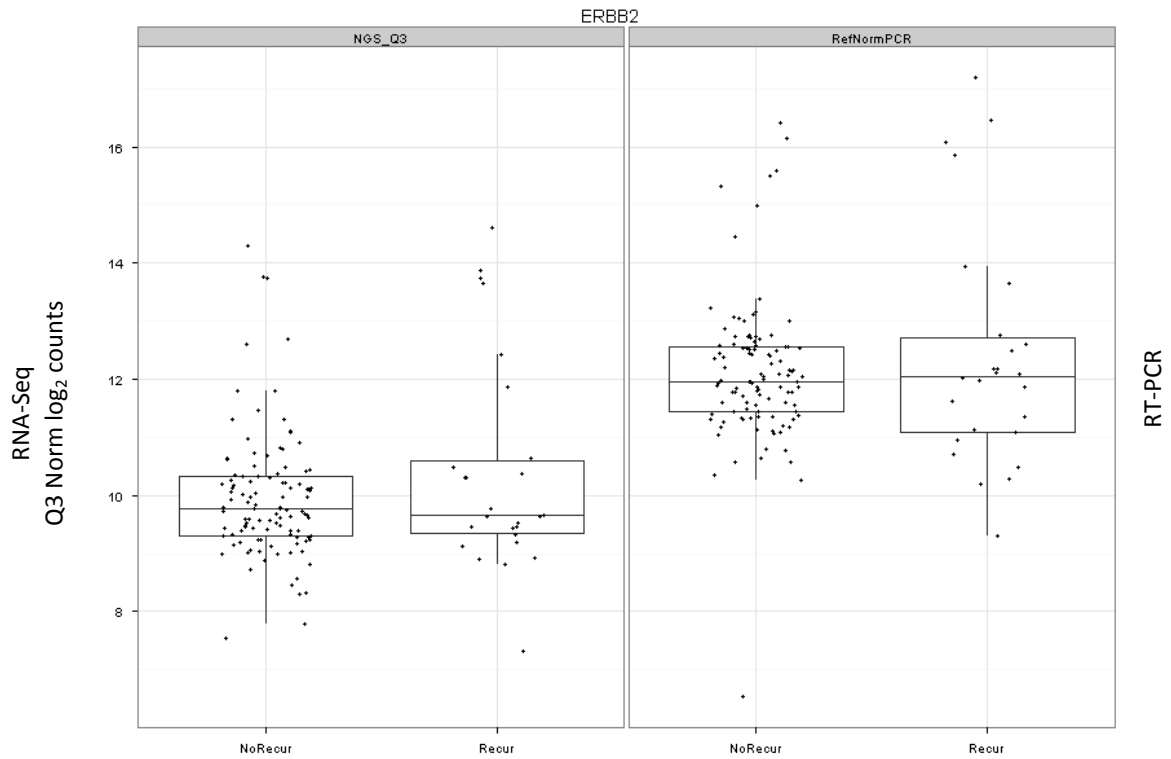

2G

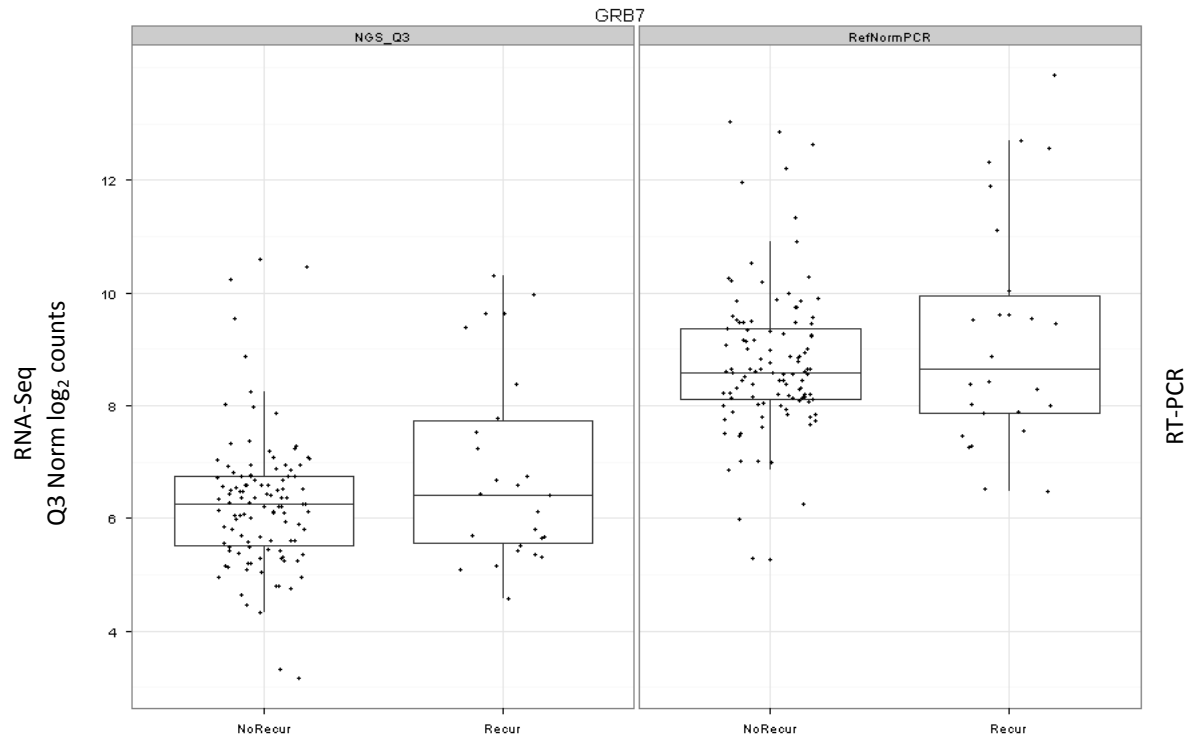

2H

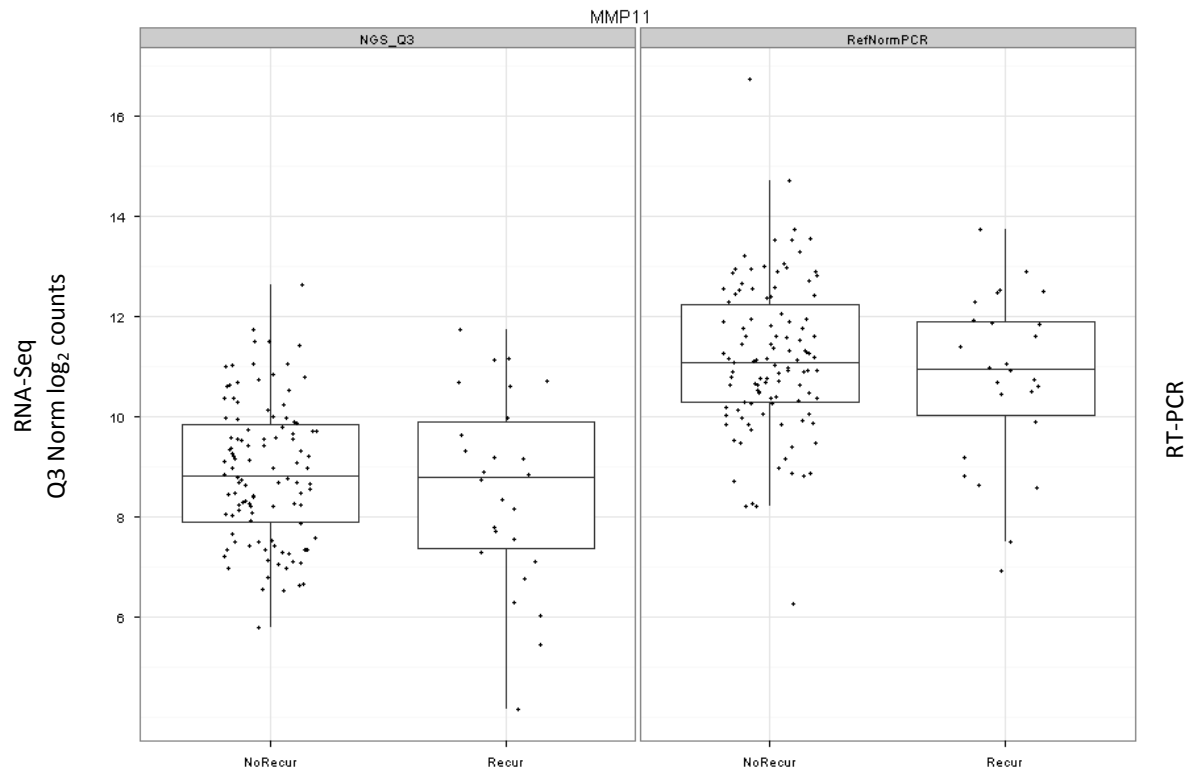

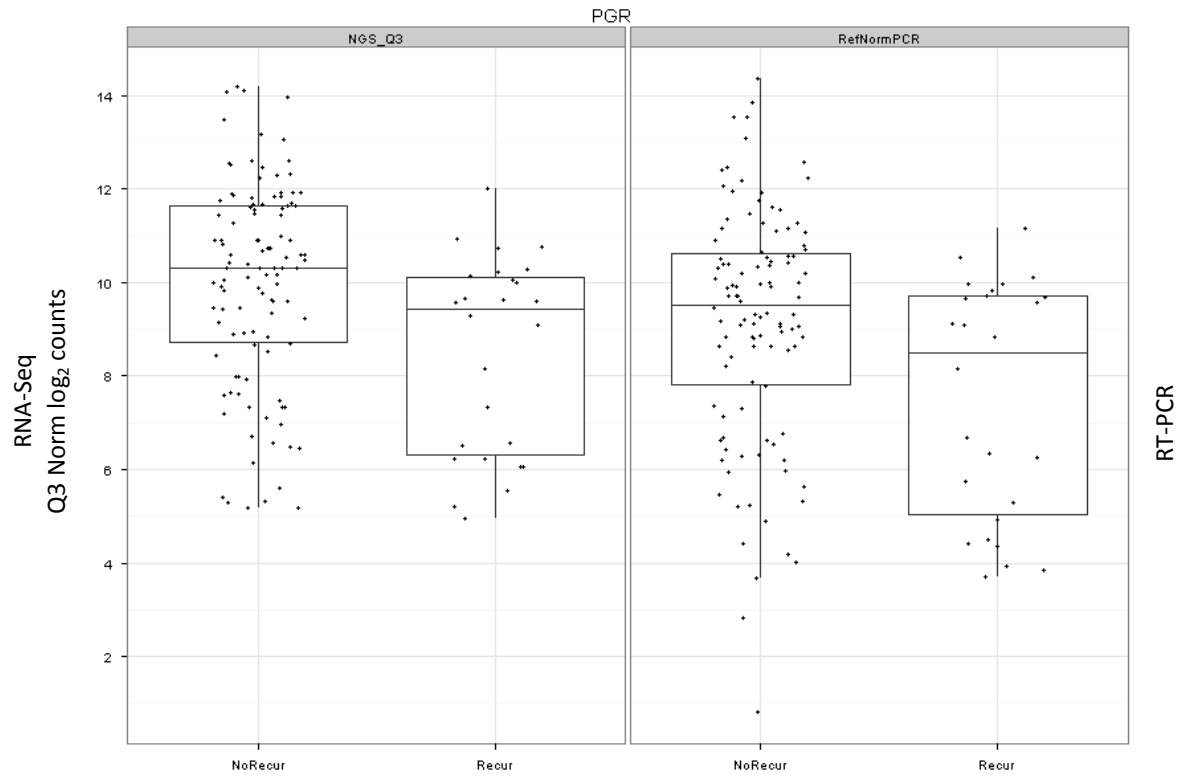

Supplement: Figure S2 — Box plots of normalized expression values of RNAs in breast cancer patients, stratified by recurrence status. Each point represents a patient tumor. The bottom and top of the box are the 25th and 75th percentiles and the horizontal band within the box is the 50th percentile (median) of the points in the group. The ends of the vertical lines represent the lowest datum still within 1.5 inter-quartile range of the lower quartile, and the highest datum still within 1.5 inter-quartile range of the upper quartile. Values from RNA-Seq (left panel) and RT-PCR (right panel) are shown. A. BAG1; B. BIRC5; C. CCNB1; D. CD68; E. ESR1; F. ERBB2; G. GRB7; H. MMP11; I. PGR. (PDF) [file pone.0040092.s002.pdf]

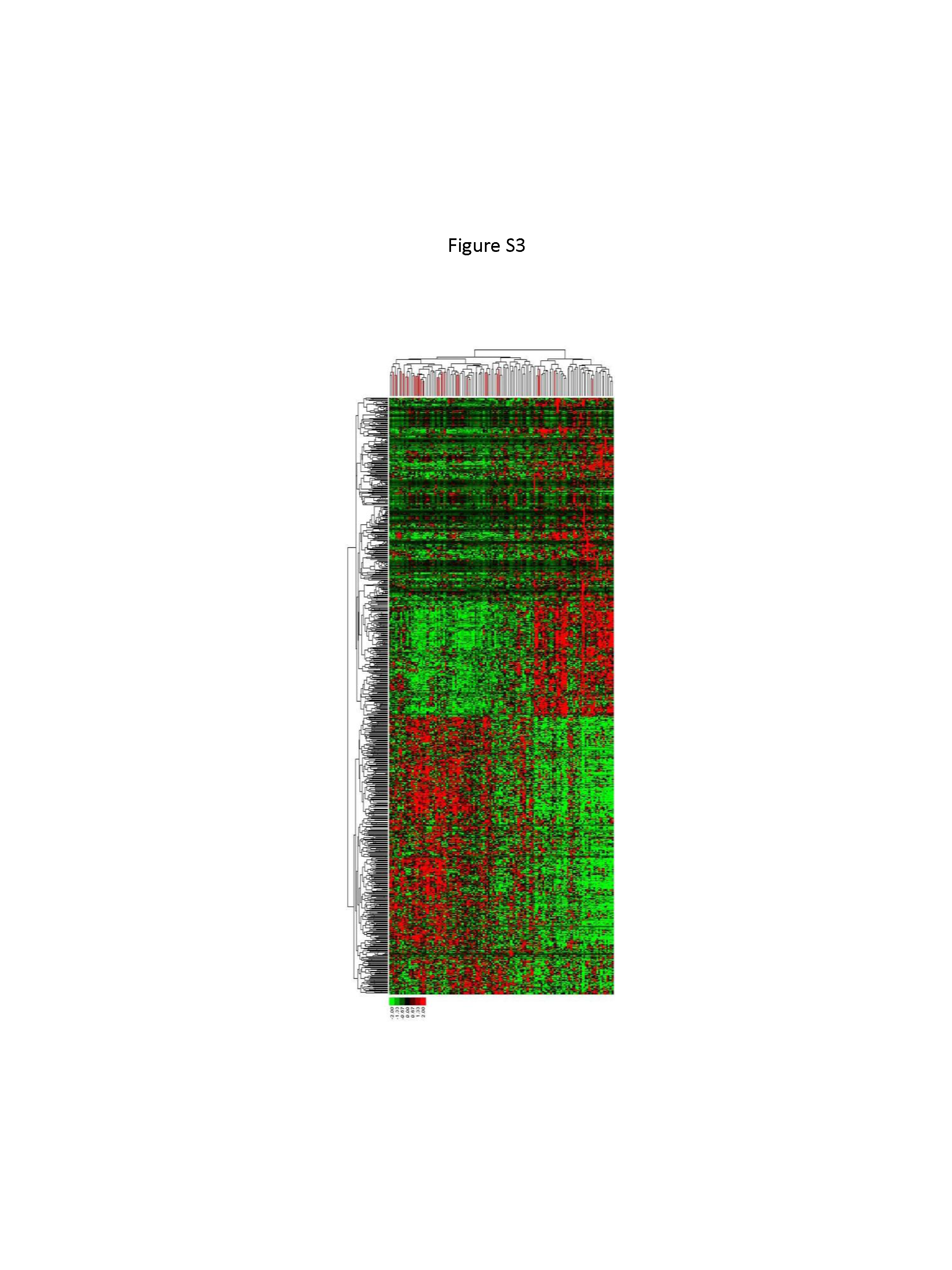

Supplement: Figure S3 — Heat map for expression of Identified RefSeq RNAs in 136 breast cancer patients. All 1307 Normalized expression values of RefSeq RNAs that were found to be associated with risk of breast cancer recurrence (Table S3) are represented on the vertical axis. Patients are represented on the horizontal axis at the top of the figure. (TIFF) [file pone.0040092.s003.tiff]
