# Supplementary material for: Whole Transcriptome RNA-Seq Analysis of Breast Cancer Recurrence Risk Using Formalin-Fixed Paraffin-Embedded Tumor Tissue
Source: PLoS One. 2012 Jul 13;7(7):e40092. doi: 10.1371/journal.pone.0040092 (PMC3396611; doi:10.1371/journal.pone.0040092)
Supplement: Method S1 — The uniquely mapped reads from all (136) patients were filtered to a depth of 1 read to eliminate potential noise from mis-mapping, and other sources. These reads were clustered into individual read islands based on the overlap of their human genome map coordinates, yielding 12,750,071 islands. Nearby islands were consolidated by a merging distance cutoff that was calculated by a maximum likelihood estimation (MLE) to maximize overlap of identified regions with known genes. This yielded a cutoff of 30 base pairs (bp) and 6,633,258 regions of interest (ROIs). These were then filtered by the three retention criteria: 1) average read count ≥5 across the tested patient population, 2) ROI length ≥100 base pairs, 3) read depth (average read number divided by the length of the ROI) ≥0.075. ROIs among the remaining 23,024 were classified as intergenic if they did not overlap with the transcripts annotated in the UCSC refFalt.txt file. A total of 2101 intergenic region ROIs were obtained from this process. (DOCX) [file pone.0040092.s012.docx]

**Method S1.** The uniquely mapped reads from all (136) patients were filtered to a depth of 1 read to eliminate potential noise from mis-mapping, and other sources. These reads were clustered into individual read islands based on the overlap of their human genome map coordinates, yielding 12,750,071 islands. Nearby islands were consolidated by a merging distance cutoff that was calculated by a maximum likelihood estimation (MLE) to maximize overlap of identified regions with known genes. This yielded a cutoff of 30 base pairs (bp) and 6,633,258 regions of interest (ROIs). These were then filtered by the three retention criteria: 1) average read count ≥ 5 across the tested patient population, 2) ROI length ≥100 base pairs, 3) read depth (average read number divided by the length of the ROI) ≥0.075. ROIs among the remaining 23,024 were classified as intergenic if they did not overlap with the transcripts annotated in the UCSC refFalt.txt file. A total of 2101 intergenic region ROIs were obtained from this process.
